# How Do Minerals, Vitamins, and Intestinal Microbiota Affect the Development and Progression of Heart Disease in Adult and Pediatric Patients?

**DOI:** 10.3390/nu15143264

**Published:** 2023-07-24

**Authors:** Peet Brecht, James Curtis Dring, Felipe Yanez, Agnieszka Styczeń, Paulina Mertowska, Sebastian Mertowski, Ewelina Grywalska

**Affiliations:** 1 Department of Cardiology, Medical University of Lublin, Jaczewskiego 8, 20-093 Lublin, Poland; 2 Department of Experimental Immunology, Medical University of Lublin, 20-093 Lublin, Poland

**Keywords:** CVD, heart disease, macronutrients, micronutrients, vitamins, minerals, microbiome, atherosclerosis, nutrients, diet

## Abstract

Cardiovascular diseases (CVDs) are the leading cause of death worldwide, far ahead of cancer. Epidemiological data emphasize the participation of many risk factors that increase the incidence of CVDs, including genetic factors, age, and sex, but also lifestyle, mainly nutritional irregularities and, connected with them, overweight and obesity, as well as metabolic diseases. Despite the importance of cardiovascular problems in the whole society, the principles of prevention of CVDs are not widely disseminated, especially among the youngest. As a result, nutritional neglect, growing from childhood and adolescence, translates into the occurrence of numerous disease entities, including CVDs, in adult life. This review aimed to draw attention to the role of selected minerals and vitamins in health and the development and progression of CVDs in adults and children. Particular attention was paid to the effects of deficiency and toxicity of the analyzed compounds in the context of the cardiovascular system and to the role of intestinal microorganisms, which by interacting with nutrients, may contribute to the development of cardiovascular disorders. We hope this article will draw the attention of society and the medical community to emphasize promoting healthy eating and proper eating habits in children and adults, translating into increased awareness and a reduced risk of CVD.

## 1. Introduction

Cardiovascular diseases (CVDs) continue to be a global health challenge, accounting for a significant number of deaths worldwide [1,2,3,4,5]. It is noteworthy that while symptoms of CVD typically manifest in adulthood, risk factors associated with the disease often emerge during childhood and adolescence [6,7,8,9]. Various factors contribute to the development of CVD, including family history [10], age and gender [11], high blood pressure [12], high cholesterol [13], smoking [14], diabetes [15,16], obesity and overweight [17,18,19], physical inactivity [20], unhealthy diet [21], and stress [22,23]. Although various factors contribute to the development of these conditions, emerging evidence suggests that our dietary choices play a crucial role in shaping cardiovascular health. The intricate relationship between nutrition and cardiovascular well-being is an ever-evolving field of study, and recent research has shed light on the remarkable influence of both micro- and macronutrients in preventing and managing CVDs. Micro- and macronutrients form the building blocks of our diet, and their interplay within the human body intricately regulates numerous physiological processes. Micronutrients, including vitamins, minerals, and trace elements, are essential for cellular function, antioxidant defense, and enzymatic reactions. Macronutrients, namely carbohydrates, proteins, and fats, serve as the main sources of energy and play vital roles in cellular structure, hormone production, and overall metabolism [24,25,26]. The balance and quality of these nutrients directly impacts cardiovascular health, contributing to the development or prevention of CVDs. While the influence of diet on cardiovascular health is well-established, recent research has unearthed an intriguing connection between micronutrients, the essential vitamins and minerals vital for physiological functions, and the gut microbiota—a complex ecosystem of microorganisms residing in our gastrointestinal tract. This evolving area of study sheds light on the interplay between micronutrients, the gut microbiota, and their impact on CVDs’ development and progression [27].

Therefore, this literature review is aimed at the demand of the human body for minerals and vitamins necessary to maintain health and their involvement in the pathogenesis of CVDs. In addition, special attention was paid to the role of intestinal microorganisms, which, directly interacting with nutrients, may contribute to the development and progression of this group of diseases, which is particularly dangerous for life.

## 2. Importance of Micronutrients and Vitamins for Human Health

**Figure 1:**
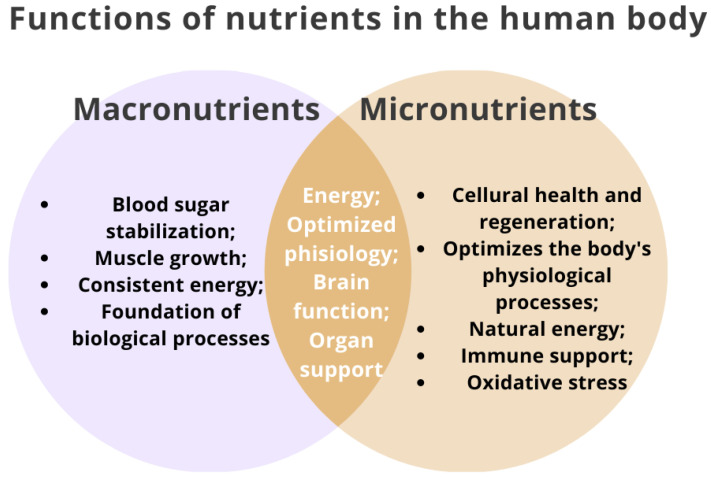
Functions of nutrients in the human body based on [32].

Micronutrients are essential nutrients that the human body requires in small amounts for proper growth, development, and overall health. Unlike macronutrients, such as carbohydrates, proteins, and fats, which are needed in more significant amounts, micronutrients are required in trace amounts [28]. Micronutrients include vitamins and minerals that play critical roles in various physiological processes. Micronutrients are essential for good health and are involved in many processes like immune function, bone health, blood clotting, nerve function, and many more (Figure 1) [24,25,26]. Eating a balanced diet that includes a variety of fruits, vegetables, whole grains, lean proteins, and dairy products can help ensure adequate micronutrient intake. In some cases, dietary supplements may be recommended to address specific deficiencies, but a healthcare professional should be consulted before starting any supplementation. All deviations from the norm, both of mineral compounds and vitamins, may indicate deregulation of the body’s functioning, affecting signaling disorders, cellular processes, and the dysfunction of organs, consequently leading to the development of pathological conditions. Therefore, early detection of changes in the concentration of macro- and micronutrients and vitamins in serum samples is a reliable indicator of a person’s homeostasis [29,30,31].

**Figure 2:**
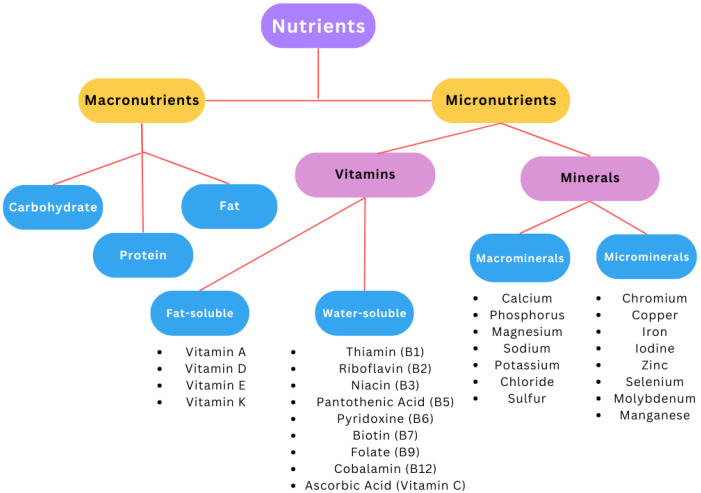
Classification of nutrients based on [33,34].

Micronutrients and macronutrients are minerals, i.e., inorganic substances involved in many critical metabolic processes. Micronutrient requirements are below 100 mg per day, while those elements with higher standards are called macronutrients (above 100 mg per day) (Figure 2). The human demand for these components complements the direction of the microenvironment and cells. Mineral efficiency can use hormonal integration, cofactors in chemical reactions, and the ability to rebuild tissue after trauma or local destruction [33,34].

**Figure 3:**
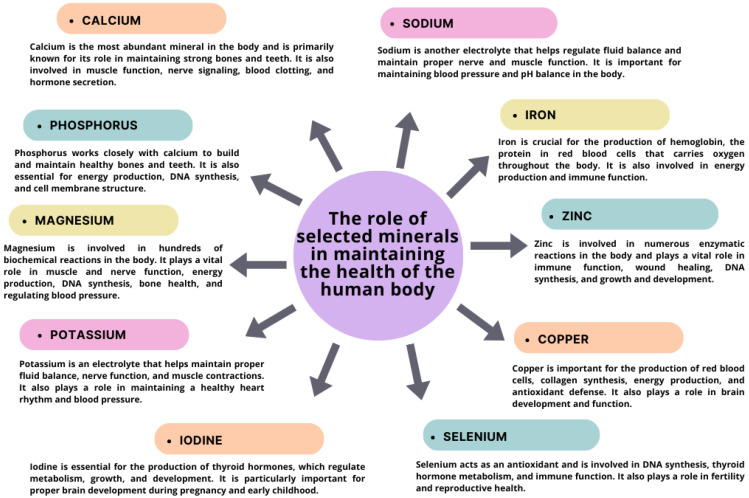
The role of selected minerals in maintaining the health of the human body based on [29,35].

Each mineral has specific functions and requirements, so it is essential to eat a balanced diet that includes a variety of nutrient-rich foods to ensure adequate mineral intake [29]. The impact of an insufficient or excessive intake of macro- and micronutrients can be a subject of debate. Health and fitness influencers are critical in navigating the constant battle between accurate and misleading information. These essential elements have a significant impact on the functioning of various organs and the body’s immunity, including cellular and humoral immunity, as well as the clinical response [29,35,36,37]. Several studies have demonstrated the vital role of various mineral compounds, including zinc, iron, selenium, copper, and vitamin, in maintaining the health of the human body (Figure 3) [38,39,40,41,42,43,44].

It should be noted that mineral deficiencies (malnutrition) and CVDs can interact to create a vicious circle. While malnutrition is often associated with inadequate food intake and starvation, its relationship with CVDs is complex and multifaceted. Malnourished individuals may experience deficiencies in key nutrients such as vitamin A, vitamin C, vitamin D, and iron, which can disrupt normal cardiovascular function, impair vascular health, and compromise immune responses. Moreover, undernutrition during critical developmental stages, such as pregnancy and early childhood, may have long-lasting effects on cardiovascular health later in life [45,46,47,48]. Solving these problems requires a multidimensional approach. Adequate nutrition through a well-balanced diet that meets the body’s needs for energy, protein, and micronutrients is essential to support immune function and contributes to reducing the risk of CVDs [49,50]. More than just supplements is needed.

Vitamins are essential organic compounds that the body requires in small amounts for proper functioning, growth, and overall health [29]. They play crucial roles in various physiological processes, including metabolism, immune function, cell growth and repair, and nerve function [29]. Vitamins are categorized into two main groups: fat-soluble vitamins and water-soluble vitamins [51,52]. Vitamin deficiencies affect all age groups and often coexist with mineral deficiencies [53]. Pregnant and lactating women and young children are particularly susceptible to vitamin deficiencies [53]. Such deficiencies affect their biochemical roles, and avitaminosis can cause clinical abnormalities [51,53,54]. However, it is worth noting that despite the lack of toxicity associated with a high intake of fortified foods [55], it is important to recognize that each year, over 60,000 individuals, including children under the age of six, experience life-threatening effects due to vitamin toxicity [55,56].

Vitamins play a significant role in cardiovascular health and can greatly influence the risk and development of CVDs [57]. Vitamins crucial to maintaining cardiovascular well-being, such as vitamin D, have been linked to calcium absorption, bone health, and immune function, all impacting cardiovascular health. Vitamin E acts as an antioxidant and helps protect cells from damage, including oxidative stress, which is a risk factor for CVD. Additionally, vitamin K is required for proper blood clotting and has implications for maintaining healthy blood vessels [51]. While the preventive effect of multivitamin use on chronic diseases among healthy individuals remains a matter of debate [57], studies have indicated that certain vitamins may have specific benefits for cardiovascular health. It is important to note that relying solely on supplements is not a substitute for a healthy lifestyle. A balanced diet rich in fruits, vegetables, whole grains, lean proteins, and low-fat dairy products, which naturally provide a variety of vitamins, remains the foundation for cardiovascular health [49,57]. Irritability, lethargy, developmental delay, bone changes, or fractures can be vitamin D deficiency symptoms in children. Despite considerable scientific evidence proving the significant impact of vitamins on human health, in the literature, we can also find publications presenting the need for more effects of using multivitamins in the prevention of chronic diseases among healthy people [57]. Another systematic review by O’Connor et al. shared convincing evidence that macronutrients and micronutrients play no significant role, except for a slight advantage in cancer incidence when using supplements; there is little or no effect on avoiding cancer, CVD, and mortality [58]. Despite these reports, doctors and specialists recommend, in some cases/people, the use of vitamins to increase functional and cellular needs.

## 3. The Role of Macro- and Micronutrients in the Course of Heart Disease in Adults

### 3.1. Importance of Calcium (Ca)

Calcium not only plays a role in contractility but also creates and regulates heart rhythm and metabolism. Therefore, considerations of calcium metabolism in the onset and persistence of heart disease cannot be limited to a specific heart disease entity. Instead, the role of calcium in many categories of heart disease, such as arrhythmia, heart failure, and ischemic heart disease, should be discussed.

The National Osteoporosis Foundation (BHOF) and the American Society for Preventive Cardiology (ASPC) concluded that the effect of calcium and vitamin D with or without vitamin D intake is not associated with CVD in the healthy adult population [4]. However, meta-analysis shows that a dietary calcium intake of 700–1000 mg/day or calcium supplementation of 1000 mg/day significantly increases the risk of cardiovascular and ischemic heart disease [59]. In the treatment of coronary artery disease, later meta-analyses have shown that calcium monotherapy is associated with an increased risk of myocardial infarction [60].

### 3.2. The Importance of Potassium (K)

There is a clear relationship between serum potassium and sodium concentrations, which is of great importance in mortality in healthy adults and may be mainly related to the increase in blood pressure. The World Health Organization (WHO) recommends a potassium intake of at least 90 mmol/d (3510 mg/d) for adults [61]. Studies have shown an inverse relationship between the amount of potassium and sodium. Teenagers with increased sodium intake have elevated blood pressure, while it can also be seen in those with low potassium intake (>7.0 mg/d) [62], although potassium supplementation has been studied to have a positive effect on both dystopia and contraction blood pressure, the exact amount is not yet known.

### 3.3. The Importance of Selenium (Se)

Participants in the National Health and Nutrition Examination Survey (NHNAS) analyzed the role of trace elements in hypertension between the ages of 8 and 80. A positive relationship was found between serum selenium and hypertension, regardless of age and antihypertensive medications; while the copper–zinc serum showed no significant results. Serum selenium levels of 120 μg/L or higher (reference level 70–150 μg/L) were significantly associated with hypertension [63]. It is crucial to highlight the significance of selenium in numerous pathways. Selenium’s antioxidative activity is believed to positively impact cardiovascular health, with possible mechanisms involving reduced oxidative stress and inflammation, as well as regulation of circulating lipid levels [64].

Other studies by NHANES have shown that elevated serum selenium levels are correlated with increases in HDL, LDL, and triglycerides. The benefits of selenium supplementation are only seen in those with current deficits, helping to lower HDL, LDL, and triglyceride levels [64,65,66].

### 3.4. Importance of Copper (Cu)

The current US and Canadian recommended dietary allowance (RDA) for Cu is 9 mg/d for adult men and women, with an upper tolerable intake level (UL) of 10 mg/day for adults [67]. While an essential micronutrient for humans, Cu is toxic in large amounts. Overcharging with this metal quickly leads to Fenton-type redox reactions, resulting in oxidative cell damage and death. This is not a problem, as the human body seems to deal with large amounts through various types of excretion and resorption of the element [68].

Serum copper is associated with total cholesterol levels in children and adolescents in the United States, although the exact mechanism by which copper contributes to total cholesterol is unknown [69]. In previous studies, a high serum copper level, with or without a low serum zinc level, was associated with cardiovascular risk, a non-significant difference between the copper-to-zinc ratio, and with the risk of hypertension [70,71]. On the opposite spectrum of serum Cu concentrations, deficiency of this element not only exacerbates changes in hypertension but also changes in cardiac morphology, including larger myocytes, myofibril abnormalities, and rupturing of the basal lamina at the capillary–myocyte interface [67].

### 3.5. Importance of Zinc (Zn)

Observations suggest that patients with idiopathic dilated cardiomyopathy exhibit higher serum Cu levels and lower serum Zn levels than healthy individuals, supporting previous findings. Zinc deficiency in the myocardium leads to significant structural and functional cell dysfunction, indicating that Zn levels could be a diagnostic and predictive marker in chronic failing cardiomyopathies [72]. Contrary to previous beliefs, high zinc intake does not offer protection against hypertension. A recent cohort study found that individuals with the highest quintile of zinc intake were less likely to have hypertension but more likely to have diabetes [70].

The association between serum Zn concentrations and CVDs has been explored in several studies. Soinio et al. (2007) [73] reported an increased incidence of cardiovascular events in patients with type 2 diabetes who had low serum zinc levels. Similarly, Suliburska et al. (2010) [74] found that lower dietary zinc intake and serum zinc concentrations were associated with a higher risk of insulin resistance, hypertension, diabetes, and hypertriglyceridemia, all CVD risk factors. Maret (2019) [75] discussed the involvement of zinc in the activated methyl cycle and its implications for CVD, highlighting elevated serum homocysteine concentrations as a CVD risk factor in Zn-deficient rats. However, zinc supplementation did not affect this increase. 

Joo et al. (2021) [76] revealed that low serum Zn levels were linked to increased mortality in patients with CVD, emphasizing the potential benefits of dietary Zn supplementation in individuals with chronic metabolic diseases. Ghasemi et al. (2014) [77] identified a weak positive association between serum Zn and high-density lipoprotein cholesterol (HDL-C) levels in women, suggesting a potential relationship between disturbances in copper and zinc homeostasis and metabolic syndrome in CVD. Rostan et al. (2002) [78] and an Indian study reported a decreased relative risk of cancer and CVD and an inverse association between dietary zinc intake, serum zinc levels, and the prevalence of coronary artery disease and diabetes. Richter et al. (2017) [79] suggested a potential inverse correlation between dietary zinc status and CVD risk, considering alcohol consumption as an important covariate in this relationship.

Choi et al. (2018) [80] summarized the evidence supporting the association between zinc deficiency and CVD development, underscoring the potential diagnostic and prognostic implications of zinc homeostasis-associated molecules in CVD. Peng & Wei (2017) [81] found that low serum zinc concentrations and high serum copper concentrations were linked to inflammatory factors in patients with CVD.

These studies provide valuable insights into the relationship between serum zinc levels and CVD risk, highlighting the potential benefits of zinc supplementation and the involvement of zinc in various physiological processes related to CVD development.

### 3.6. The Importance of Iron

A recent meta-analysis showed that iron and dietary supplements such as vitamin B6, vitamin A, multivitamins, and antioxidants have no apparent significant effect on mortality or CVD outcomes [82]. However, some texts shed light on the benefits of iron in the heart failure population, demonstrating benefits in left ventricular ejection fraction after supplementation [83]. The same favorable result can be said for those with heart failure with reduced ejection fraction (HFrEF), in whom intravenous iron, as opposed to its oral counterpart, had an average 8.8-point improvement on the Kansas City Cardiomyopathy Questionnaire (KCCQ) after six months [84]. An overall analysis of iron therapy has been associated with better quality of life parameters, fewer hospitalizations, and increased exercise capacity in the treatment treating heart failure [85]. In heart failure, patients who are iron deficient, ferric carboxymaltose supplementation may improve symptoms, functional capacity, and overall health. In addition, ferric carboxymaltose reduces the incidence of recurrent hospitalizations due to CVD [86,87].

### 3.7. Importance of Magnesium

Although government measures have been taken in many Western countries, magnesium deficiency is commonly observed in the population. It is estimated that 56–68 percent of Americans do not consume enough magnesium per day to cover the recommended daily allowance [88]. The literature suggests a lower daily minimum magnesium intake of 350 mg for men and 280–300 mg for women [89]. Recent meta-analyses have confirmed that both serum and dietary magnesium deficiencies have a significant increase in the risk of CVD and coronary heart disease (CHD). Moreover, both serum magnesium and dietary magnesium are inversely and linearly associated with the risk of CVD and CHD, meaning that higher levels of this mineral may lead to health benefits in patients with CVD/CHD [90]. It is also important to determine magnesium’s ability to improve vascular tone and endothelial function while reducing platelet aggregation and the risk of stroke [91,92]. All these properties lead to an associated lower risk of coronary heart disease by up to 22% when adequate amounts of magnesium are taken in the diet [93].

## 4. The Role of Vitamins in the Pathogenesis of Heart Disease in Adults

### 4.1. Fat-Soluble Vitamins

#### 4.1.1. Vitamin A—Retinol

The health benefits of vitamin A are numerous, but its impact on the cardiovascular system has been a topic of controversy. Some studies have indicated that the consumption of vitamin A does not correlate with the incidence of hypertension [94,95], although research indicates that vitamin A is involved in endothelial function by altering nitric oxide pathways [96]. In addition, vitamin A also acts as an anti-inflammatory agent, and a lack of it can cause or exacerbate inflammation [97].

Zhang et al. found that in 12,245 people in a 6.1-year cohort study, found that those who consumed less vitamin A (<227.3 mg RE/day) compared to those who consumed more vitamin A (>227.3 mg RE/day) were significantly less likely to develop hypertension. Both plant-based and animal-based vitamin A intakes have been shown to produce comparable results [98]. The same group of researchers found a significant inverse correlation between plasma retinol and the risk of having a first stroke in hypertensive Chinese adults [99].

#### 4.1.2. Vitamin D

It is estimated that one billion people worldwide are deficient in vitamin D, making this a significant public health problem on a global scale [100,101,102,103]. Vitamin D helps control the renin-angiotensin-aldosterone system (and consequently blood pressure), vascular cell development, obesity, energy expenditure, pancreatic cell activity, and inflammatory and fibrotic pathways [104,105]. Vitamin D deficiency is associated with vascular dysfunction, arterial stiffness, left ventricular hypertrophy, and hyperlipidemia [104]. Numerous types of immune cells, including platelets, macrophages, dendritic cells, and the three major types of cardiovascular cells (vascular smooth muscle cells, endothelial cells, and cardiomyocytes), have been found to contain vitamin D receptors (VDRs) [106]. Regardless of blood 25(OH)D levels, studies have shown that age is generally associated with lower levels of vitamin D receptor (VDR) expression [104]. Vitamin D passes through the cell membrane and cytoplasm to enter the nucleus where it binds to the VDR. When this complex binds to the retinoic acid receptor, it changes the way they work, which starts the process of making proteins.

The liver produces a glycoprotein with a molecular weight of 58 kDa, which binds to vitamin D. It is the primary transporter of calcitriol. Its job is to activate macrophages, get rid of action and bind fatty acids, which helps vitamin D get to the tissues it needs to reach. Polymorphisms in proteins that bind vitamin D may alter their binding to vitamin D. This may be directly related to the risk of vitamin D deficiency or CVD [106]. Vitamin D deficiency in humans is associated with vascular dysfunction, arterial stiffness, and left ventricular (LV) hypertrophy. Deficiency of VDR leads to disturbance of homeostasis, cardiac metalloproteases, and fibroblasts, which results in an increase in the left ventricle (LV) and elevated levels of atrial natriuretic peptide. This, in turn, stimulates the production of a fibrous extracellular matrix, leading to left ventricular (LV) dilatation and reduced electromechanical coupling [107]. Due to the factors mentioned above, vitamin D has been; however, there is also much debate about this: in a double-blind, placebo-controlled RCT, healthy people took 800 IU of cholecalciferol daily for 12 weeks to see if it can lower blood pressure, heart rate, and other risk factors for CVD. The results showed that vitamin D did not reduce serum CVD risk markers [108].

A recent meta-analysis examined the relationship between serum 25(OH)D levels and CVD risk for the first time. The study included 25 prospective cohort studies of 10,099 cases conducted between April 2000 and September 2017. No significant association was found, but lower vitamin D levels were associated with a 44% higher relative CVD risk (incidence and death combined) and higher CVD mortality (RR = 1.54, 95% CI: 1.29–1.84) [109]. The Vitamin D Assessment (ViDA), a double-blind, placebo-controlled study, was designed to test whether giving high doses of vitamin D once a month to the general population could help prevent CVD. Thus, over 5000 people in New Zealand took vitamin D3 orally or with a placebo. They started with a dose of 200,000 IU and then received a dose of 100,000 IU every month for a median of 3.3 years. Approximately 25% were not getting enough vitamin D. The primary measure of CVD incidence was 11.8% of those in the vitamin D group and 11.5% of those in the placebo group, whether or not they were vitamin D deficient at baseline. The conclusion was that taking a high dose of vitamin D once a month does not prevent CVD [110].

#### 4.1.3. Vitamin E

The first evidence of the action of α-tocopherol (vitamin E) showed that it inhibited the activity of protein kinase C and subsequently slowed down the growth of smooth muscle cells in rat aortic smooth muscle (A7r5) and human smooth muscle (HAI) cell lines. Moreover, treatment of human SMCs, HL-60 macrophages and THP-1 monocytes with alpha-tocopherol has been shown to inhibit the cellular uptake of oxidized low-density lipoproteins (ox-LDL) by downregulating CD36 expression [111,112,113]. Other forms of vitamin E, such as tocotrienols, have been shown to reduce CVD risk by lowering blood cholesterol and triglyceride levels, which are important risk factors for CVD [114]. Tocotrienols may affect cholesterol metabolism by decreasing the oxidation of LDL and inhibiting the production of 3-hydroxy-3-methylglutaryl-coenzyme, which is a reductase enzyme that plays a crucial role in cholesterol production [115]. These findings, combined with results from animal studies, support the concept that tocotrienols have a preventive function in the progression of atherosclerosis [116,117]. Due to their ability to activate proteasomes and improve myocardial function, tocotrienols have been identified in several studies as cardioprotective molecules [118].

In addition, further studies have compared the effects of vitamin E supplementation against food intake. Ehab et al. found that a higher intake of fat-soluble vitamins (K, E, and D) was associated with a lower risk of death from heart failure in Japanese women but not men [119]. According to the results of an unavailable scientific article, women who took vitamin E supplements for more than two years had a 41% lower risk of developing CVD compared to controls [120,121]. In intervention studies, Loffredo et al. found that taking vitamin E supplements alone reduced the risk of myocardial infarction, and taking vitamin E supplements combined with other antioxidants appeared to have no effect [122]. Controversially, a recent Mediterranean diet study found no correlation between vitamin E and overall mortality in people at high cardiovascular risk [123]. Similarly, the Heart Outcomes Prevention Evaluation study found that 400 IU. α-tocopherol taken daily for 4–6 years did not improve cardiovascular outcomes in a cohort of high-risk elderly patients [124]. Vitamin E is a key nutrient and many studies have shown an inverse correlation between vitamin E levels and diseases such as CVD, and some studies have shown no beneficial results which may be due to too many different limitations such as too little absorption and not enough dosage. As a recommendation for future double-blind studies placebo-controlled with 400 IU, 800 IU, placebo (as a control), and vitamin E in the diet, may bring some light to research without significant results.

#### 4.1.4. Vitamin K

In addition to other vital functions, vitamin K acts as an anti-inflammatory modulator. In a cross-sectional study of 1381 participants, plasma phylloquinone levels and intake were shown to be adversely associated with circulating markers of inflammation such as CD40 ligand and IL-6 [125]. A cross-sectional study of 662 community residents showed that higher blood levels of phylloquinone were associated with multiple markers of serum inflammation, including IL-6, soluble intercellular adhesion molecule-1 (ICAM-1), and CRP [126]. Thus, the anti-inflammatory effect of vitamin K on vascular cells may prevent inflammatory vascular disorders such as atherosclerosis and vascular calcification. Vitamin K has been suggested to help avoid formation of coronary artery calcifications (CACs) due to the inhibitory effect of matrix Gla proteins (MGP-vitamin K_2_-dependent, Gla-containing MGPs) on mineral deposition in the arteries [127,128]. When MGP is activated by vitamin K-dependent carboxylation and serine phosphorylation, it strongly inhibits arterial calcification [129]. In addition, it has been proposed that pro-inflammatory cytokines such as TNF-α may play a significant role in the development of medial calcifications in diabetes and CKD [130].

### 4.2. Water-Soluble Vitamins

#### 4.2.1. Vitamin B3—Niacin

In addition to the more well-known pellagra disease caused by vitamin B3 deficiency, which is still prevalent in undeveloped countries [131], niacin (1000–3000 mg/day, up to 6000 mg/day) significantly lowers plasma cholesterol, lowers LDL cholesterol, and increases cholesterol HDL, while reducing mortality due to ischemic heart disease [132]. Studies have shown that the agonistic effect of NA on the nicotinic acid receptor GPR109A is a Gi-class A-linked rhodopsin-like GPCR responsible for this agonistic effect throughout the body. The GPR109A receptor is expressed in white and brown adipose tissue, spleen, and immune cells such as macrophages, monocytes, dendritic cells, and neutrophils [133,134]. Niacin has been linked to anti-atherosclerotic effects in addition to its impact on LDL/HDL. In both adipocytes [135] and “non-foamed” macrophages [136], B3 activates the GPR109A receptor, leading to upregulation of the peroxisome proliferator-activated receptor (PPAR) and liver X receptor (LXR) transcription factors. PPAR and LXR have been shown to promote the transcription of the ATP-binding cassette transporter proteins A1 (ABCA1) and G1 (ABCG1), which remove cholesterol from the bloodstream in vitro and ex vivo [137,138]. Niacin may exert its antiatherosclerotic effects in atherosclerotic lesions in two possible ways, by stimulating the removal of cholesterol from macrophage foam cells and by stimulating reverse cholesterol transport [139,140], given these findings and evidence that GPR109A expression in peripheral macrophages has been established.

#### 4.2.2. Vitamin B6—Pyridoxine

Vitamin B6 administration decreased IL-6 levels and increased total lymphocyte counts in people with chronic inflammation, a critical mechanism driving the development of atherosclerosis [141]. One of the most widely studied effects of vitamin B6 is its effect on CVD and blood pressure. Low plasma PLP levels in humans are associated with a high risk of atherosclerosis, stroke, and thrombosis [142,143]. In a large prospective cohort study, Jeon and Park found that higher intakes of vitamin B6 in Korean men reduced the risk of CVD but not in Korean women [144]. Another study showed that postmenopausal women with lower fasting concentrations of activated vitamin B6 had a higher chance of developing myocardial infarction (MI) [145].

The latest analysis of the literature by Kumurgsee indicates that vitamin B6 helps to protect the heart through the action of histamine, GABA, and imidazole dipeptides but also contributes by regulating the P2 inflammasome 7R-NLRP3196. In addition, modulation of anserine, carnosine, histamine, and GABA, as well as the P2X7R-NLRP3 inflammasome, may significantly decrease inflammation and oxidative stress [146].

#### 4.2.3. Vitamin B12 and B9 (Folic Acid)

A deficiency of vitamins B12 and B9 may result in elevated total homocysteine (tHcy) in the blood, which is associated with the progression of CVD [147,148,149,150]. Folic acid and vitamin B12 supplementation reduced blood homocysteine levels by 25 percent and 7 percent, respectively, in a meta-analysis of 12 randomized controlled trials [151]. There are different ways that homocysteinemia can increase the risk of ischemic stroke. Oxidative stress, protein homocysteinylation, and Ca^2+^ dysregulation are closely related to the dysfunction of nerve cells and the blood–brain barrier caused by excess homocysteine. Homocysteine may further enhance apoptosis, neuronal death, and dysregulation of the blood–brain barrier by inducing hypomethylation of deoxyribonucleic acid [152]. An intact vascular endothelium is critical to avoid cardiovascular sequelae such as ischemic stroke, and elevated homocysteine levels can cause endothelial dysfunction in several pathways. This includes increased oxidative stress, decreased nitric oxide bioavailability, increased endothelial inflammation with vascular adhesion molecules and leukocyte recruitment, which promotes platelet activation and thrombosis [153,154]. These implications of defective endothelium exacerbate all phases of ischemic stroke, from early atherosclerosis to thrombosis. This is a key factor contributing to the additional risk of ischemic stroke in people with high homocysteine levels, as well as those with CVD. In observational studies, there is a correlation between blood THC levels and the risk of coronary heart disease [155]. However, this association has yet to be replicated in current and recent Mendelian randomized trials [156,157]. According to a meta-analysis, folic acid supplementation has been shown to reduce the incidence of stroke by 10% and overall CVDs by 4%. Participants with no history of CVD benefited most from the study. In randomized controlled trials, folic acid supplementation did not reduce the incidence of coronary artery disease. On the other hand, folic acid has been shown to increase the flow-mediated vasodilating function of the endothelium as a surrogate measure of CVD risk in various clinical intervention studies. In various clinical intervention studies, B9 supplements have also been reported to increase flow-mediated endothelial vasodilatory function as a surrogate measure of CVD risk [158]. Given this contrast, it is likely that total homocysteine (tHcy) is a marker of CHD risk rather than a causative factor. In particular, prospective randomized clinical trials in large populations are needed to better define the function, optimal route of administration, and contribution of dietary supplementation with other antioxidant ingredients. To obtain standardized and reproducible data, evaluating the importance of vitamin B9 and B12 in cardiovascular health and disease will be necessary.

#### 4.2.4. Vitamin C—Ascorbic Acid

Vitamin C is essential in many processes related to the pathophysiology of CVDs. However, when interpreting the results of studies on the impact of vitamin C on human health, significant limitations should be considered. For example, dietary assessment methods such as food questionnaires and food diaries need to be more precise and accurate, and consider conditions that may affect vitamin C levels and homeostasis. In addition to the well-studied severe vitamin C deficiency, also known as scurvy, low vitamin C levels have been linked to high blood pressure, endothelial dysfunction, heart disease, atherosclerosis, and stroke [159,160,161]. According to Baker et al. [162], vitamin C supplementation significantly increased endothelial-derived NO synthase activity in human umbilical vein endothelial cells. A meta-analysis of 14 studies involving 374,488 participants with a 10-year follow-up showed that dietary vitamin C was associated with the risk of CHD. Still, supplement use is not significantly associated with CHD risk [163]. Dietary vitamin C intake, as measured by a food frequency questionnaire, was associated with a lower risk of CHD death in Japanese women with no history of CVD or cancer [164]. Another meta-analysis of 15 studies involving 188,209 participants showed that vitamin C supplementation ranging from 120 to 1000 mg per day in combination with vitamin E and beta-carotene had no decisive effect on major cardiovascular events or ischemic heart disease [165]. Comparing the results of the European Investigation into Cancer and Nutrition (EPIC)—Norfolk, a long-term study of 20,299 healthy people, it is clear that blood samples provide a more accurate measure of vitamin C levels than food intake questionnaires. Ascorbate will be associated with a lower risk of heart disease after a follow-up of 12.8 years; for every 20 μmol/L increase in plasma vitamin C, the risk of HF [166] was reduced by 9%. Vitamin C supplementation, according to a meta-analysis of eight controlled trials involving 15,445 people, showed no significant association with major cardiovascular events or cardiovascular death [167].

Between 1990 and 2017, a systematic review and meta-analysis by Ran et al. found that the concentration of vitamin C in the blood was significantly lower in hypertensive patients than in normotensive people; additionally, the authors identified a robust inverse relationship between serum vitamin C levels, SBP, and DBP [168]. Similar results were reported in another systematic review and meta-analysis by Jajedi et al. in 2019, confirming the inverse relationship between ascorbic acid and blood pressure [169].

Vitamin C increases oxidative imbalance and vascular remodeling induced by various stressors such as reperfusion injury after myocardial infarction [170], hyperoxia [171], stress [172], prolonged immobilization [173], glucose load [174], psychological stress [175], and significantly impairs endothelial barrier permeability [176,177], an aspect that has significant implications in infectious disorders [178,179], which are also known to cause a systemic increase in oxidative stress [180,181].

## 5. The Importance of Macro- and Micronutrients and Vitamins in the Development and Progression of Heart Disease in Pediatric Patients

Mineral deficiencies in children can significantly impact their overall health and may indirectly affect their long-term cardiovascular health. Few literature studies indicate the significant role of iron, zinc, calcium, and vitamin D deficiencies in pediatric patients.

Iron deficiency is one of the most common nutritional deficiencies worldwide, especially among children. Iron is essential for the production of red blood cells and the transport of oxygen throughout the body. Severe iron deficiency can lead to anemia, affecting cardiovascular health by reducing oxygen delivery to tissues and increasing stress on the heart. Chronic anemia in children is associated with increased cardiovascular risk, including hypertension and impaired exercise capacity [182,183,184,185].

Zinc plays a crucial role in numerous physiological processes, such as immune function, growth, and development. It is indispensable for the proper functioning of approximately two hundred distinct enzymes, many of which are involved in the synthesis of DNA and RNA. An estimated 17.3% of the world’s population does not consume enough zinc. Insufficient zinc levels can hinder the immune system, increasing vulnerability to infections. In children, chronic conditions can lead to systemic inflammation, causing endothelial dysfunction, atherosclerosis, and ultimately elevating the risk of CVD [186,187,188,189].

Calcium and vitamin D deficiencies often go hand in hand because vitamin D is required for proper absorption and utilization of calcium [190,191]. Insufficient calcium and vitamin D intake in childhood can affect bone development and increase the risk of diseases such as rickets. Although the direct link between childhood calcium/vitamin D deficiency and CVDs is not fully understood, evidence suggests that poor bone health in childhood may carry over into adulthood and contribute to cardiovascular risk factors such as hypertension and dyslipidemia [192,193].

It should be noted that mineral deficiencies can often be prevented through a balanced and nutrient-rich diet. Promoting a healthy diet that includes a variety of mineral-rich foods such as lean meats, dairy products, legumes, whole grains, and fruits and vegetables is critical to meeting children’s nutritional needs. When deficiencies are suspected or diagnosed, health professionals may recommend specific interventions, such as dietary changes or supplementation, to address weaknesses and mitigate potential long-term health risks.

## 6. Exploring the Significance of Intestinal Microbiota in the Development and Progression of Heart Disease

**Figure 4:**
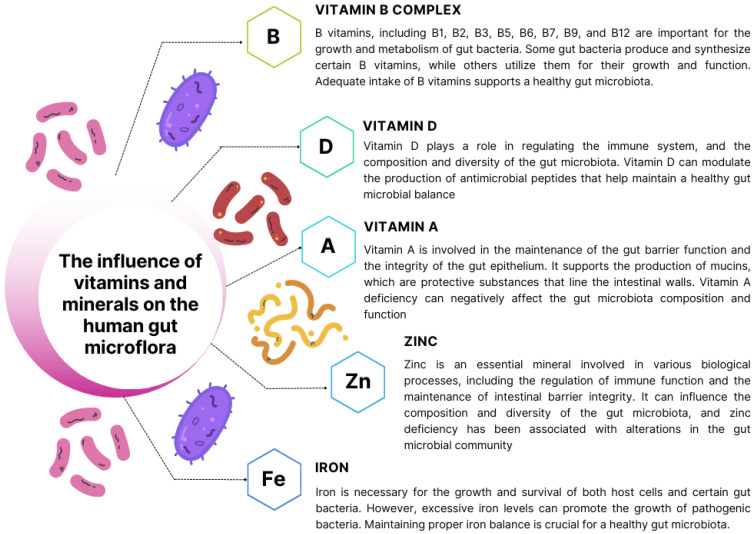
The influence of selected vitamins and minerals on the human intestinal microflora based on [196,197,198,199,200,201,202,203,204,205].

The significance of intestinal microbiota in the development and progression of heart disease is a growing area of research. Vitamins and minerals play crucial roles in maintaining overall health and supporting the function of various bodily systems, including the gut microbiota. The gut microbiota encompasses a diverse community of microorganisms, including bacteria, viruses, fungi, and other microbes, residing in the gastrointestinal tract. These microorganisms interact with the host and play vital roles in digestion, immune function, metabolism, and nutrient absorption [194,195]. Ongoing research is aimed at elucidating the effects of vitamins and minerals on the functioning of intestinal microorganisms, as depicted in Figure 4.

**Figure 5:**
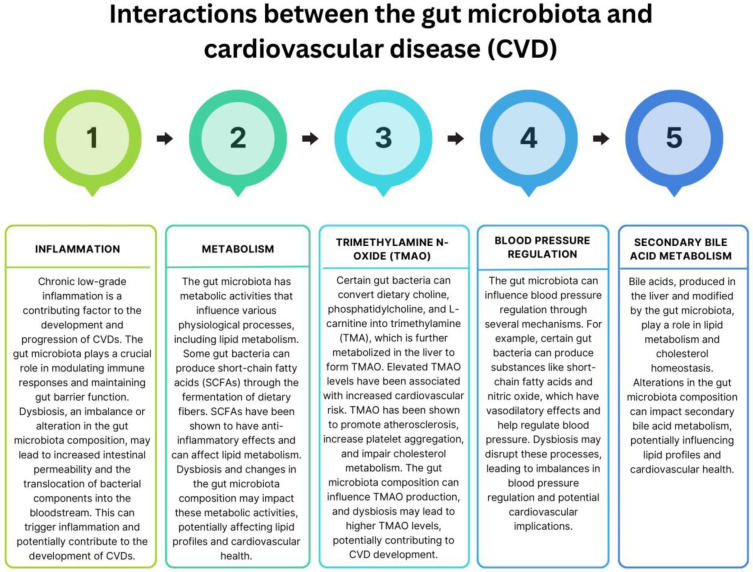
Interactions between the gut microbiome and CVD based on [206,207,208,211,212,213,214,215,216].

It should be noted that the relationship between vitamins, minerals, and gut microbiota is complex and multifaceted, necessitating further investigation. While these nutrients have the potential to influence the gut microbiota, the composition of the gut microbiota itself can also impact the absorption and metabolism of various vitamins and minerals. Moreover, emerging evidence suggests that dysbiosis of the intestinal microbiota is implicated in the pathogenesis of numerous diseases, including CVD [206,207,208,209,210]. Recent studies indicate a potential link between the composition and abundance of the complex microbial community in the digestive tract and CVD. Although the precise mechanisms and causation are not fully understood, research has revealed some connections and interactions between the gut microbiome and CVD, as illustrated in Figure 5.

In-depth investigations conducted by Qi Zhu in adult patients with coronary artery disease (CAD) have successfully identified dysbiosis, dysfunctions, and entire networks of intestinal microflora [217]. These findings suggest that targeted modifications aimed at the gut microbiota could present an innovative approach to CAD treatment. In children, gut microbiota and nutrition have been unequivocally demonstrated as essential components in the development of severe acute malnutrition [218]. Dysbiosis has also been associated with various conditions affecting both children and adults, including autism, attention deficit hyperactivity disorder, asthma, and allergic reactions [219]. A recent longitudinal study involving over 900 neonates demonstrated that the mode of delivery and the infant’s gut microbiota, particularly members of the *Lachnospiraceae* family, mediated the association between maternal overweight before pregnancy and obesity in children between 1 and 3 years of age [220]. Furthermore, infants with low levels of Bifidobacterium spp. and high levels of Staphylococcus aureus were found to be more likely to develop obesity by the age of 7 [221]. Notably, microbial genes involved in vitamin synthesis, novel folic acid synthesis, and amino acid metabolism are more abundant in children compared to genes associated with inflammation in adults [222].

Considering a prophylactic approach to pre- and probiotics during adolescence could potentially benefit the hereditary aspects of CVD and aid in the prevention of type 2 diabetes. Several immune-mediated diseases and metabolic disorders, such as diabetes and obesity, have been implicated in the pathogenesis of host-microbiota interactions involving inflammatory and metabolic pathways. The mounting body of research suggests that alterations in the gut microbiota may play a role in the development of CVD. Recent advancements in technology have allowed for a better understanding of the microbiome and its various functions. Targeting the microbiome using pre- and probiotics holds promise based on studies involving human participants, although larger-scale trials are needed to establish these correlations [210]. It is worth noting that these findings differ from previous animal models that demonstrated reduced infarct size and improved post-infarction cardiac function with oral vancomycin or a combination of antibiotics targeting the microbiome [223,224]. The scale of conflict in CVD treatment is unprecedented, and careful consideration is required to address global concerns regarding bacterial resistance and its impact on microbial communities.

By exploring the intricate interplay between vitamins, minerals, and gut microbiota, future insights may provide novel strategies for managing and preventing CVD.

## 7. Conclusions and Research Perspectives for the Future

Evidence-based techniques in dietary health offer solutions for prevention, providing individualized nutritional treatment for children and adolescents based on their developmental stages. However, implementing these interventions is challenging due to the time and cost required to develop new approaches for reducing childhood obesity. Involvement of patients, families, healthcare sectors, communities, and governments is recommended to address juvenile obesity and its association with heart disease. Practical health and nutrition messages play a vital role in building healthy habits and lifestyle changes during the current public health crisis.

Understanding the human gut microbiota is crucial for growth, development, and overall health promotion. Ongoing research aims to unravel its diverse functions and identify therapeutic targets in various diseases and metabolic disorders. Exploring the specific genes and enzymes that influence the gut microbiota’s impact on health and disease is an important area of investigation. Additionally, investigating how certain foods and vitamins activate or deactivate specific criteria is crucial. Further efforts should be made to enhance preventative care, with a focus on overlooked environmental causes, to reduce and prevent cardiovascular conditions. Multidisciplinary meetings and personalized nutritional treatment based on specific phases of transformation hold potential in managing hereditary diseases in children and adolescents. 

While the development of additional techniques to reduce overall obesity requires attention and investment, future research should continue to narrow down the health and nutrition benefits and determine optimal amounts of vitamins and micronutrients for personalized approaches. Leveraging technological advances, such as microenvironment testing and utilizing the gut microbiome, is promising for use in personalized medicine. Providing accurate information to healthcare professionals is crucial in achieving disease prevention, promoting optimal health, and reducing cardiovascular conditions.

## Data Availability

Not applicable, the information provided in the manuscript is based on a literature review of available scientific articles.
